# Optimal Channel Design: A Game Theoretical Analysis

**DOI:** 10.3390/e20090675

**Published:** 2018-09-05

**Authors:** MHR. Khouzani, Pasquale Malacaria

**Affiliations:** School of Electronic Engineering and Computer Science, Queen Mary University of London, Mile End Road, London E1 4NS, UK

**Keywords:** entropy, game theory, convex optimisation, quantitative information flow

## Abstract

This paper studies the problem of optimal channel design. For a given input probability distribution and for hard and soft design constraints, the aim here is to design a (probabilistic) channel whose output leaks minimally from its input. To analyse this problem, general notions of entropy and information leakage are introduced. It can be shown that, for all notions of leakage here defined, the optimal channel design problem can be solved using convex programming with zero duality gap. Subsequently, the optimal channel design problem is studied in a game-theoretical framework: games allow for analysis of optimal strategies of both the defender and the adversary. It is shown that all channel design problems can be studied in this game-theoretical framework, and that the defender’s Bayes–Nash equilibrium strategies are equivalent to the solutions of the convex programming problem. Moreover, the adversary’s equilibrium strategies correspond to a robust inference problem.

## 1. Introduction

A channel is defined as a conditional distribution, modelling the probability of outputs that an adversary can observe given secret inputs. Important examples of channels are side-channels in computer security where an attacker, for example by observing the running time of an encryption program, can reconstruct the encryption keys.

At a high level, the problem of optimal channel design is the following: given a prior on the secret and some operational constraints, design a channel that minimises the leakage of information about the secret. In simple terms, an optimal channel can be seen as an optimal countermeasure to information leakage.

To explore this design problem, one needs to specify what constraints should be considered and how the leakage of information is quantified. In the cryptographic example above, one may want, for example, to design a channel of minimal leakage (in terms of the number of key bits that can be reconstructed by an adversary) under the constraint that the average encryption per block should take less time than some given duration. This work will consider two general classes of constraints which we refer to them as “hard” and “soft”. Hard constraints are the ones establishing which outputs are allowed given each inputs. These constraints must be satisfied for each realisation of input–output pairs. Soft constraints, on the other hand, must be satisfied in the expected value sense, as they relate to the expected utility of the channel.

Leakage of information is defined as the difference between the adversary’s prior and posterior uncertainty, i.e., the uncertainty before and after observing the outputs of the channel. The leakage quantifies how much the attacker can learn about the secret input from observing the outputs. Therefore, any entropy function, seen as a measure of uncertainty, can induce a candidate function for quantifying leakage. For Shannon entropy, the leakage is just the mutual information. To capture the widest class of entropies, and hence leakages, this work uses core-concavity [1,2], a generalisation of concavity which allows for capture of entropies which are not concave (like Rényi entropies when α>1).

Once the optimal channel design problem is formally set, it is possible to address some basic questions. The first question regards how difficult it is to solve this problem. Based on Reference [2], it can be shown that, for any choice of the entropy measure, this problem is solved via convex programming with zero duality gap, for which the Karush–Kuhn–Tucker (KKT) conditions can be used to solve for the optimal channel.

### 1.1. Literature Review

The problem of information leakage outside of the communication setting has been studied in the quantitative information flow (QIF) literature [3,4,5,6], works on private information retrieval (PIR) [7], and private search queries [8,9], as well as research on privacy-utility trade-offs [10,11,12]. Particularly important from the field of QIF are advances on fundamental security guarantees of leakage measures (what security can be achieved) and robust techniques and results (how much a technique or result is valid across different notions of leakage). However, most of the theoretical effort has been focused on analysing a given system as opposed to a design problem.

Information leakage in the context of game theory has been studied in Reference [13]. Their work focuses on modelling the interplay between attacker and defender in regard to information leakage of given channels, and to reason about their optimal strategies. In contrast our focus is on the design of optimal channels within operational constraints.

The authors in Reference [14] also use a zero-sum game between a forecaster against Nature to show that the celebrated maximum entropy principle in statistics, i.e., that one should choose a distribution that has the highest entropy from a family without any further knowledge, is the dual of solving a robust Bayes decision problem. This was the inspiration for our duality connection in Section 5.2.

This work builds and extends on our two conference papers [1,2]. However, there are several differences compared to those papers. For example, we have now simplified the definition of core-concavity without loss of generality. In addition, the games in Reference [1] are different; e.g., they do not include soft constraints. Moreover, the connection of convex optimisation to a two-person game for “any” core-concave entropic leakage was not explored in either works. Finally, the relation of the dual problem, that of the adversary, to a robust information extraction problem is unique to this manuscript.

### 1.2. Contributions

The main contribution of this paper is to present the problem of designing optimal channels for minimum information leakage in a game-theoretical framework for a generalised class of quantifying leakage. In this way, the optimal channel design can be studied both from the defender (the channel designer) and the adversary (the inference maker, or the information extractor) point of view. The main technical contribution is Theorem 1, which shows that the convex programming solutions as in Reference [2] correspond to the defender’s optimal strategies in these games. Moreover, this game-theoretical framework reveals that there is a tight duality relationship between the problem of designing a minimal leakage channel and choosing a “robust inference extraction” strategy. In particular, knowing only the specification of a channel given by some constraints and a prior distribution, the optimal strategy to extract the maximum amount of information about the input from the output of the channel, where the exact realisation of the channel is unknown, needs to be found. Hence, the strategy should be robust to any realisation of the channel within its constraints. When the game is finite, efficient solutions for both the defender and adversary’s strategies can be found using linear programming.

This work also establishes a result to deal with uncertainty about the prior. By Theorem 2, it follows that, when the prior is not unique, but is known to depend on a hidden “context”, the Nash equilibrium is not given by customising with respect to the context, but rather by treating the multi-prior problem as a single-prior one, where the prior is the average prior over all contexts.

### 1.3. Roadmap

After introducing notations and the information-theoretical background, including the important definitions of core-concavity and gain functions, the optimal channel design problem is presented in Section 3. It is then shown, in Section 4, that the problem is solved by convex programming for any entropy belonging to this generalised class.

The main contribution of the paper, i.e., the game-theoretical framework, is presented in Section 5. The games under study here are two persons sequential zero-sum games with asymmetric information. A notion of utility is introduced based on gain functions and soft constraints and the saddle-point equilibria are defined. The main result of this section, Theorem 1, shows the correspondence between equilibria and convex optimisation from Section 4. The section concludes with a discussion of the problem from the adversary point of view and its relation to robust inference.

In Section 6, our framework is extended to the case of uncertainty about the prior. It is first analysed as a convex optimisation problem, culminating in Theorem 2, which is followed by a discussion of the game-theoretical implication of that result.

## 2. Notational Conventions and Preliminaries

We will denote sets, random variables, and realisations with calligraphic, capital, and small letters, respectively, e.g., X, *X*, and *x*. We will denote the cardinality of a set X by |X|. For a vector *p*, we use $p_{\left[i\right]}$ to denote the *i*-th largest element of *p*, where ties are broken arbitrarily. In addition, we will use the notation ${∥p∥}_{\alpha }$ for the α-norm of vector *p*, that is, ${∥p∥}_{\alpha }:={\left(\sum_{i=1}^{n}p_{i}^{\alpha }\right)}^{1/\alpha }$. The limit case of ∞-norm is ${∥p∥}_{\infty }:=p_{\left[1\right]}$.

Let *X* represent the *secret* as a discrete random variable that can take one of the *n* possibilities from X:={1,…,n} with the (categorical) distribution of $p_{X}=\left(p_{X}\left(1\right),p_{X}\left(2\right),\ldots ,p_{X}\left(n\right)\right)\in p\left(X\right)$, where p(X) is the probability simplex in $R^{n}$. For the rest of the paper, as is the convention, we may omit the subscript *X* whenever it is not ambiguous and simply use *p* to refer to $p_{X}$. Without loss of generality, assume that every secret has a strictly positive probability of realisation and that p(x)’s are sorted in non-increasing order; that is, p(1)≥p(2)≥…≥p(n)>0.

A system that generates *observable Y* from the discrete set Y that can probabilistically depend on a secret can be modelled as a probabilistic discrete channel (henceforth referred to simply as a “channel”) denoted by the triplet $\left(X,p_{Y|X},Y\right)$. Specifically, X and Y are the *input* and *outputalphabets*, respectively, and $p_{Y|X}$ denotes the conditional probability distribution, also known as the transition matrix. That is, p(y|x) is the probability with which the channel produces the output (the observable) *y* given that its input (the secret) is *x*. In particular, they satisfy the following:

(1a) $$\begin{array}{c} p(y|x)\geq 0\;\;\;\;\;\forall x\in X,\;y\in Y; \end{array}$$

(1b) $$\begin{array}{c} \sum_{y\in Y}p\left(y|x\right)=1\;\;\;\forall x\in X. \end{array}$$

In other words, the transition matrix is “row-stochastic”. In the rest of the paper, we will use the terms secret and input, as well as observables and outputs interchangeably.

Central to this work is the notion of leakage of information. In order to define leakage formally we will start by defining entropy and posterior (conditional) entropy in a general context.

### 2.1. Entropy

The classical choice for entropy and posterior entropy are (*Gibbs*)–*Shannon*’s:

(2a) $$\begin{array}{c} H\left(X\right)=-\sum_{x\in X}p\left(x\right)\log\left(p\left(x\right)\right) \end{array}$$

(2b) $$\begin{array}{c} H\left(X|Y\right)=-\sum_{y\in Y^{+}}p\left(y\right)\sum_{x\in X}p\left(x|y\right)\log\left(p\left(x|y\right)\right) \end{array}$$

where $Y^{+}$ is the set of outputs that have a strictly positive probability of realisation, that is $Y^{+}=\left\{y\in Y|\exists x\in X,p\left(y|x\right)>0\right\}$. In addition, p(y) is the (total) probability that *y* is observed by the adversary, i.e., $p\left(y\right)=\sum_{x^{\prime }\in X}p\left(x^{\prime }\right)p\left(y|x^{\prime }\right)$, and p(x|y) is the *posterior probability* of the secret *x* given that *y* is observed as given by the *Bayes’ rule*: p(x|y)=p(x,y)/p(y)=p(x)p(y|x)/p(y).

However, as we mentioned in the introduction, there are many candidates for entropy. Some are more fitting for specific operational scenarios, such as Min-entropy and guesswork. A generalisation of Shannon and Min-entropy is the Rényi family, which itself is a special case of the Kolmogorov–Nagumo family. Rather than taking a specific entropy, we construct a general entropy from an axiomatic description.

Consider a random variable *X* whose distribution depends on the realisation of a “context” *C*, which is a binary random variable. In particular, p(c=0)=α and p(c=1)=1−α, with 0≤α≤1; moreover, $p_{X|c=0}=p_{1}$ and $p_{X|c=1}=p_{2}$. Compare the following two scenarios: (1) we observe the realisation of the context and (2) we cannot see the realisation of the context. Intuitively, our uncertainty about *X* in the first scenario should be lower than that in the second. In particular, if we measure the uncertainty of a random variable with distribution *p* by function F(p), we should have $\alpha F\left(p_{1}\right)+\left(1-\alpha \right)F\left(p_{2}\right)\leq F\left(\alpha p_{1}+\left(1-\alpha \right)p_{2}\right)$; that is, *F* should be a *concave* function. However, we note that this intuitive inequality still holds even if an increasing R→R function η(·) is applied to both sides; that is,

$$\begin{array}{c} \eta \left(\alpha F\left(p_{1}\right)+\left(1-\alpha \right)F\left(p_{2}\right)\right)\leq \eta \left(F\left(\alpha p_{1}+\left(1-\alpha \right)p_{2}\right)\right). \end{array}$$

The function η can be thought of as capturing our risk attitude. This motivates the following definitions.

**Definition** **1.** *Let H be a function from probability distributions to R. Then we call H to be core-concave if we can write H(p)=η(F(p)), where η:R→R is strictly increasing and F is concave*.

Throughout the paper, we will consider concave functions to also be continuous; specifically, their value on the boundaries are their limit values. Note that any concave function is also core-concave, by simply taking η(t)=t. However, the converse is not true. A notable example is the Rényi entropies:

$$\begin{array}{c} H\left(p\right)=\frac{1}{1-\alpha }\log\sum_{i}p_{i}^{\alpha }=\frac{\alpha }{1-\alpha }\log{∥p∥}_{\alpha }. \end{array}$$

For α>1, this function is neither concave nor convex (it is only pseudo-concave). However, it is core-concave. This can be shown as follows:

$$\begin{array}{c} \eta \left(t\right)=\frac{\alpha }{1-\alpha }\log\left(-t\right),\;F\left(p\right)=-{∥p∥}_{\alpha }. \end{array}$$

For 0<α<1, core-concavity can be shown by $\eta \left(t\right)=\frac{\alpha }{1-\alpha }\log\left(t\right)$ and $F\left(p\right)={∥p∥}_{\alpha }$. As another example, consider Sharma–Mittal entropies [15], defined as

(3) $$\begin{array}{c} H_{\alpha ,\beta }\left(p\right)=\frac{1}{\beta -1}\left(1-{\left({∥p∥}_{\alpha }^{\alpha }\right)}^{\frac{1-\beta }{1-\alpha }}\right),\;\alpha \geq 0,\;\alpha ,\beta \neq 1. \end{array}$$

This family generalises Rényi $H_{\alpha ,\beta \to 1}\left(p\right)$, Shannon $H_{\alpha \to 1,\beta \to 1}\left(p\right)$), and Havrda–Tsallis entropies [16,17]: $H_{\alpha ,\alpha }\left(p\right)=\frac{1}{1-\alpha }\left(1-{∥p∥}_{\alpha }^{\alpha }\right)$. $H_{\alpha ,\beta }\left(p\right)$ is also core-concave. This can be seen by

$$\begin{array}{c} \eta \left(t\right)=\frac{1}{\beta -1}\left(1-t^{\frac{1-\beta }{1-\alpha }}\right),\;F\left(p\right)={∥p∥}_{\alpha }^{\alpha }. \end{array}$$

In this paper, we take any function that is core-concave as a candidate for entropy.

### 2.2. Posterior Entropy

Motivated by the equivalence of our core-concave entropies with generalised induced entropies, we define the posterior entropy to take the following form:

(4) $$\begin{array}{c} H\left(p_{X|Y}\right)=\eta \left(\sum_{y\in Y^{+}}p\left(y\right)F\left(p_{X|y}\right)\right). \end{array}$$

Note that the above definition is deliberately *different* from $\sum_{y\in Y^{+}}p\left(y\right)H\left(p_{X|y}\right)$. In particular, η is outside of the expectation. Now, the (information) leakage can be defined as

(5) $$\begin{array}{c} \mathrm{Leakage}=H\left(p_{X}\right)-H\left(p_{X|Y}\right). \end{array}$$

The above structure of the posterior entropy is strongly motivated by the following result:

**Proposition** **1.** *For any core-concave H, leakage is non-negative*.

**Proof.**  Replacing from definitions, we have

$$\begin{array}{c} \mathrm{Leakage}=H\left(p_{X}\right)-H\left(p_{X|Y}\right)=\eta \left(F(p_{X})\right)-\eta \left(\sum_{y\in Y^{+}}p\left(y\right)F\left(p_{X|y}\right)\right). \end{array}$$

For a core-concave *H*, *F* is concave; hence, following Jensen’s inequality, $\sum_{y\in Y^{+}}p\left(y\right)F\left(p\left(x|y\right)\right)\leq F\left(\sum_{y\in Y^{+}}p\left(y\right)p_{X|y}\right)=F\left(p_{X}\right)$. Therefore, since η is a monotonically increasing function, we have $\eta \left(\sum_{y\in Y^{+}}p\left(y\right)F(p_{X|y})\right)\leq \eta \left(F\left(p_{X}\right)\right)$, i.e., leakage is non-negative. ☐

In fact, our leakages satisfy a stronger property:

**Proposition** **2.** *The conditional entropy defined in Equation *(4)* satisfies the data-processing inequality (DPI)*.

**Proof.**  Reference [1] (Lemma 1). ☐

### 2.3. Gain Functions and g-Leakage

An alternative foundational approach to information leakage is in term of gain functions. As we will use gain functions in our results, we give here a primer on this approach.

A classical interpretation for Shannon entropy is in terms of guessing a secret by asking set membership questions (“is the secret in set *X*?”). Often in the security community, another guessing model is more appropriate, which is individual guesses: “is the secret *x*”?

Information-theoretically, the individual guesses scenario is modelled by Min-entropy. This guessing scenario is, however, an *all-or-nothing* scenario: the attacker either guesses the secret or does not, and right guesses always yield the same reward. In many real world scenarios, however, even guessing part of the secret may be valuable, or guessing different secrets may yield different rewards. These scenarios have motivated the introduction of gain functions and *g*-vulnerability [18].

A gain function is a real valued function *g* whose arguments are an attacker guess and the secret: g(a,x) quantifies the gain of the attacker for guessing *a* when the secret is *x*.

The *g*-vulnerability is defined as the attacker expected gain for an optimal guess:

$$V_{g}\left(p\right)=\underset{a\in A}{\sup}\sum_{x\in X}p\left(x\right)g\left(a,x\right)$$

where A is a countable set (the attacker guesses). From *g*-vulnerability, one can define posterior *g*-vulnerability by considering the average vulnerability over all possible outputs, i.e.,

$$V_{g}\left(p_{Y|X}\right)=\sum_{y\in Y}p\left(y\right)V_{g}\left(p_{X|y}\right).$$

Further derived notions are *g*-entropy and *g*-leakage. *g*-entropy is defined as the negative log of the vulnerability: $-\log V_{g}\left(p\right)$. Similarly, posterior *g*-entropy is defined as the negative log of the posterior vulnerability: $-\log V_{g}\left(p_{Y|X}\right)$. *g*-leakage is the difference between the *g*-entropy and the *g*-posterior entropy. An important property of gain functions, which we use in the game-theoretical analysis, is that any convex function can be defined using gain functions ([19] (Theorem 5)).

## 3. Optimal Channel Design

The general setting in our paper is the following: Given a prior distribution on input (secret) variable *X* as *p*, we (the defender) would like to design a channel $p_{Y|X}$ within some operational constraints, such that the channel leaks minimally about the secret *X* through its output *Y*.

Let Ω⊆X×Y define the permissible outputs (observable) for each input (secret). Specifically, if (x,y)∉Ω, then, for input *x*, the designer cannot produce output *y*. This can represent the “hard” operational constraints on the channel. Hence, the channel, along with Equation (1), should satisfy:

(6) $$\begin{array}{c} p(y|x)=0\;\forall (x,y)\notin \Omega . \end{array}$$

We will refer to Equation (6) as “hard” constraints, as they strictly forbid some input–output pairs “path-wise”, that is, for each realisation of the input. As a consequence, an adversary can eliminate the forbidden inputs for an observable when making an inference. For ease of notation, for any given Ω, we will denote the space of channels that satisfy Equations (1) and (6) by Γ. That is,

$$\begin{array}{c} \Gamma ≜\left\{p\left(y|x\right)|p\left(y|x\right)\geq 0,\;\sum_{y:(x,y)\in \Omega }p\left(y|x\right)=1\;\forall x\right\}. \end{array}$$

The design requirement for a legitimate channel that satisfies the hard constraints can now be expressly represented as $p_{Y|X}\in \Gamma$.

The naming of hard constraints is to contrast with the “soft” constraints, which are expressed in terms of an expected value. In particular, there are many interesting cases where it may be “feasible” to assign the same observable for all secrets, but such a move may result in a huge deterioration in the system’s quality of the service (QoS). In such cases, the goal is to strike an optimal “balance” between information leakage and QoS. This is for instance the setting in geo-location privacy-utility trade-off [10,11,20] and secrecy-delay trade-off in bucketing as a defence against timing attacks [21,22].

In its most basic form, the QoS can be captured as an expected value of a “payoff” (desirability) function. In particular, let u:X×Y→R, where u(x,y) represents how good the realised output is for a particular input. Then the expected value of the pay-off is simply: $\sum_{x,y}p\left(x\right)p\left(y|x\right)u\left(x,y\right)$, which can be a metric for the QoS of the channel. The channel design problem then becomes a “two-objective” optimisation: (a) minimising leakage and (b) maximising the QoS. The solution concept for multi-objective optimisations is of “Pareto-efficiency” (Pareto-optimality), which are the solutions with a guarantee that no alternative can simultaneously improve all of the objectives (at least one of them strictly). One of the standard methods of converting a multi-objective optimisation (MOO) to (a series of) single-objective optimisations (SOOs) is to present all but one of the objectives as inequality constraints. Specifically, we can introduce a lower threshold $u_{\min}$ on the QoS by imposing: $\sum_{x,y}p\left(x\right)p\left(y|x\right)u\left(x,y\right)\geq u_{\min}$. Then by varying the value of $u_{\min}$ and solving the resulting SOOs, the Pareto-frontier (the set of Pareto-optimal solutions) will be found (see e.g., [23]). Hence, with this in mind, for the rest of the paper, we will be dealing with SOOs. We will refer to the constraint of $\sum_{x,y}p\left(x\right)p\left(y|x\right)u\left(x,y\right)\geq u_{\min}$ as the “soft” constraint, since it is expressed in terms of the expected value, distinguishing it from the “hard” constraints represented by Ω (or equivalently, Γ), for each realisation of the secret.

As we argued before, the aim is to design channels that have the lowest leakage of information about the input while satisfying a set of operational constraints, and the leakage is defined as the difference between the posterior and prior entropies. The first point to note is that the choice of the channel cannot change the prior entropy, as the prior entropy of the input is entirely governed by its prior distribution, which we assume is a “given” parameter that the defender cannot control. Therefore, the problem of minimising the leakage becomes equivalent to maximising the posterior entropy (equivocation).

Putting things together, the optimal channel design problem in its most general form becomes

(7) $$\begin{array}{c} \begin{array}{cc} \mathrm{Given}: & p_{X},\Gamma ,\eta ,F,u,u_{\min}\\ \mathrm{Solve}: & \max_{p_{Y|X}\in \Gamma }H\left(X|Y\right)=\eta \left(\sum_{y\in Y^{+}}p\left(y\right)F\left(p_{X|y}\right)\right),\;s.t.\;\sum_{x,y}p\left(x\right)p\left(y|x\right)u\left(x,y\right)\geq u_{\min} \end{array} \end{array}$$

where the main notations are described in Table 1.

**Figure 1:**
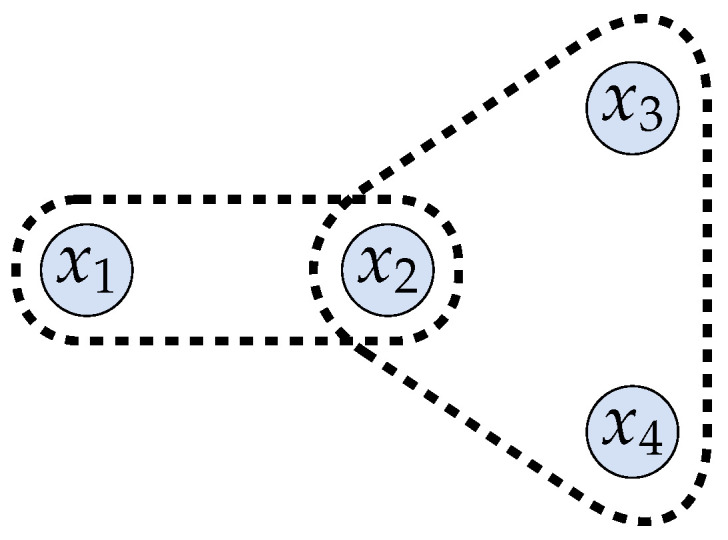
(Toy Example 1) The “secret” is one of the four possible locations $x_{1}$ to $x_{4}$. $x_{1}$ is located too far away from $x_{3}$ and $x_{4}$ for all of the secrets to be able to produce the same observable. To avoid clutter, only two of the feasible observables, $\left\{x_{1},x_{2}\right\}$ and $\left\{x_{2},x_{3},x_{4}\right\}$, are demarcated here.

Before we get to our analysis, we present two minimalistic examples to instantiate the constraints. Note that each of these contexts of course have their idiosyncrasies that are abstracted away for the purpose of this paper. The first toy example is motivated by geo-location privacy. Figure 1 depicts four locations $x_{1}$ to $x_{4}$, where the configuration is a representation of their relative positions. The defender is in one of these four locations and generates an observable, which can be its reported coordinates, based on which it receives a location-based service (LBS). Suppose, in particular, that $x_{1}$ and $x_{2}$ are near enough that the same observable can be reported for both of them, but $x_{1}$ is too far from $x_{3}$ and $x_{4}$ such that reporting the same coordinates with them is either infeasible (e.g., it will not get any network connectivity from an access point) or it will be unacceptable (the quality of the received utility will be too poor). Moreover, $x_{2}$, $x_{3}$, and $x_{4}$ are close enough to produce the same observable. If we label the observables simply by the subset of the secrets that can produce them, then the set of admissible secret-observable pairs, i.e., Ω, is $\{(x_{1},\left\{x_{1}\right\}),$ $(x_{2},\left\{x_{2}\right\}),$ $(x_{3},\left\{x_{3}\right\}),$ $(x_{4},\left\{x_{4}\right\}),$ $(x_{1},\left\{x_{1},x_{2}\right\}),$ $(x_{2},\left\{x_{1},x_{2}\right\}),$ $(x_{2},\left\{x_{2},x_{3}\right\}),$ $(x_{3},\left\{x_{2},x_{3}\right\}),$ $(x_{3},\left\{x_{3},x_{4}\right\}),$ $(x_{4},\left\{x_{3},x_{4}\right\}),$ $(x_{2},\left\{x_{2},x_{3},x_{4}\right\}),$ $(x_{3},\left\{x_{2},x_{3},x_{4}\right\}),$ $\left(x_{4},\left\{x_{2},x_{3},x_{4}\right\})\right\}$. This Ω determines the hard constraints on the problem, e.g., we must have $p(\left\{x_{2},x_{3},x_{4}\right\}|x_{1})=0$ because $(x_{1},\left\{x_{2},x_{3},x_{4}\right\})\notin \Omega$.

**Figure 2:**
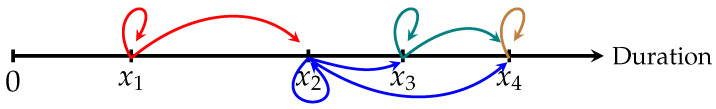
(Toy Example 2) The “secrets” are one of the four processes each with a distinct execution time $x_{1}$ to $x_{4}$. The arrows denote which process can be deferred to be released at a later finishing time. For instance, Process 2 can be either released instantaneously, i.e., at $x_{2}$, or deferred until $x_{3}$, or until $x_{4}$. In contrast, $s_{1}$ cannot be deferred as late as $x_{3}$ or $x_{4}$.

As another example, consider a minimalistic bucketing example depicted in Figure 2. The axis denotes time duration, and $x_{1}$ to $x_{4}$ represent the distinct execution times of four distinct (encryption or decryption) processes, i.e., Process 1 takes $x_{1}$ time to finish, and so on. If the result of each process is released immediately upon finishing, then they can be uniquely identified just by the timing “side channel”. The result of a finished process can be deferred and released at a later time, to become identical to other processes that take longer to finish. This superset duration time constitutes a *bucket*. In the figure, the arrows represent whether a secret can be deferred till the finishing time of a longer processes. Specifically, suppose that the delay limitation for Process 1 does not allow it to be released as late as $x_{3}$ or $x_{4}$. Therefore, the hard constraints can be identically represented as in the previous toy example.

## 4. Optimal Channel Design is Convex Programming

We now show that the problem of finding an optimal channel is a “convex optimisation” (also known as “convex programming” [24,25]). This is a useful result, because convex optimisations have desirable characteristics, e.g., many efficient algorithms for solving them exist (e.g., interior methods [25]). Moreover, any local optimum has the guarantee to also be a global optimum, so in particular any “descent” algorithm will necessarily converge to a global optimum. Additionally, in Proposition 4, we show that the Karush–Kuhn–Tucker (KKT) conditions fully describe the optimal channel (represent necessary and sufficient conditions of optimality).

**Proposition** **3.** *The optimisation problem of Equation *(7)* for any choice of the pay-off and core-concave entropy functions is solved by convex programming*.

**Proof.**  η from Equation (7) can be simply ignored for both cases, since it is an increasing R→R function. Our optimisation variable is $p_{Y|X}\in R^{\left|X||Y\right|}$. In particular, consider it as a |X||Y|×1 vector. All we need to show is that (a) the constraints of the optimisation define a convex subset of $R^{\left|X||Y\right|}$ and (b) the objective function of the maximisation is concave in $p_{Y|X}$.

Establishing (a) is simple: the constraint $p_{Y|X}\in \Gamma$, which is equivalent to Equations (1a,b) and (6) trivially define a convex subset. The minimum expected utility constraint is also linear in $p_{Y|X}$, where the coefficient of p(y|x) is p(x)u(x,y). Hence, the constraints of the problem define a convex subset of $R^{\left|X||Y\right|}$. In fact, they define a bounded polyhedron, as the feasible set is the intersection of half-spaces and it does not contain a whole line.

We establish part (b) by expressing *H* as a composition of a number of transformations that preserve concavity:

- First affine transformation $f_{i}$: projection of p(x,y) onto the sub-coordinate where $y=y_{i}$, that is, the transformation ${\left(p\left(x_{j},y_{i}\right)\right)}_{i,j}\to {\left(p\left(x_{j},y_{i}\right)\right)}_{j}$. Composition with an affine mapping preserves concavity/convexity.
- Second affine function $g_{1}$: extension of a vector with its summation of elements, i.e., the transformation: $x\to (x,\sum_{j}x_{j})$.
- Perspective transformation $g_{2}$: Given a function $F:R^{n}\to R$, consider $g_{2}:R^{n+1}\to R$, called *perspective* transformation, defined as follows: $g_{2}\left(y,t\right)=tF\left(y/t\right)$ where $\mathrm{dom}\;g_{2}=\left\{\left(p,t\right)|p/t\in \mathrm{dom}\;F,\;t>0\right\}$. Then, if *F* is concave, so is $g_{2}$ [24] (Chapter 3.2.6).

Now, we can write

$$\begin{array}{c} H\left(p\left(x,y\right)\right)=\sum_{i:p(y_{i})>0}p\left(y_{i}\right)F\left(p(x|y_{i})\right)=\sum_{i:p(y_{i})>0}g_{2}\left(g_{1}\left(f_{i}\left(p\left(x,y\right)\right)\right)\right). \end{array}$$

Hence, *H* is concave in p(x,y). ☐

As mentioned before, a fundamental property of convex optimisations is that any local optimum is a global optimum. In what follows, we establish another important property of the optimal channel design problems: that the Karush–Kuhn–Tucker (KKT) conditions provide both necessary and sufficient conditions for optimality. For an overview of the Lagrangian duality and KKT conditions the reader can consult with the rich literature on convex programming such as [24] (Chapter 5) and [26] (Chapter 28).

**Proposition** **4.** *KKT conditions are necessary and sufficient for solving the optimal channel design problem described by Equation* (7).

**Proof.**  We start by noticing that, in the most basic form, KKT conditions are expressed for cases where the function in the objective and constraints are “continuously differentiable”, whereas some of our convex objective functions (e.g., in the case of min-entropy or guesswork) are piecewise linear. There is however a simple and standard translation from piecewise-linear convex functions into continuously differentiable functions by forming the epigraph problem [24] (§5.2.5).

The proof is straightforward: all of our constraints are affine hence the KKT conditions are necessary—this is known as “Linearity Constraint Qualification” (LCQ). Moreover, since we showed that these problems are convex optimisations, the KKT conditions are also sufficient [24] (§5.5.3). ☐

The “Lagrangian” for the problem of Equation (7), denoted by *L* is:

(8) $$\begin{array}{c} L=\sum_{y}\left[\sum_{x^{\prime }}p_{x^{\prime }}p\left(y|x^{\prime }\right)\right]F\left(\frac{{\left(p_{s}p\left(y|x\right)\right)}_{x\in X}}{\sum_{x^{\prime }}p_{x^{\prime }}p\left(y|x^{\prime }\right)}\right)+\sum_{x,y}\lambda_{y}^{x}p\left(y|x\right)+\sum_{x}\mu_{x}\left(\sum_{y}p\left(y|x\right)-1\right)+\;\;\;\\ \rho \left(\sum_{x,y}p_{x}p\left(y|x\right)u\left(x,y\right)-u_{\min}\right)+\sum_{(x,y)\notin \Omega }\gamma_{y}^{x}p\left(y|x\right) \end{array}$$

where the multipliers μ,γ are from the equality constraints and are therefore free (no sign constraint), whereas the multipliers λ,ρ pertain to inequalities constraints and are hence required to be positive (dual feasibility).

The optimisation problem then becomes equivalent to solving the following KKT conditions:

1. Vanishing first order derivatives of *L* with respect to each of the optimisation variables p(y|x), that is, ∇L=0 (where ∇ is the gradient with respect to the (primal) variables p(y|x)). That is, for each p(y|x): $\frac{\partial L}{\partial p(y|x)}=0$.
2. Primal feasibility: $p_{Y|X}\in \Gamma$.
3. Dual feasibility:$\lambda_{y}^{x}\geq 0,\;\forall x,y$, and ρ≥0.
4. Complementary slackness: $\forall x,y\;\lambda_{y}^{x}p\left(y|x\right)=0$ and $\rho (\sum_{x,y}p_{x}p\left(y|x\right)u\left(x,y\right)-u_{\min})=0$.

## 5. Game-Theoretical Interpretation

We now present a game-theoretical framework for the general optimal channel design problem. The problem solution is shown to be a Nash equilibrium in a sequential zero-sum game. The main result proved in this section is a correspondence between any defender Nash equilibrium in these games and convex programming problems from Proposition 3. Moreover, when the game is finite, the solution can be found with linear programming and, hence, in a more efficient way than the general case. An important property of the game interpretation is that it provides not only the optimal channel design but also the attacker optimal attack strategy.

Consider the following two-player zero-sum game between a defender and an adversary: “Nature” chooses a realisation of a random variable *X* from the finite set X according to the publicly known probability distribution *p*. The defender, observes the realisation of *x*, and chooses an action from the finite set Y. Hence, the space of the *pure* strategies of the defender are all functions from X to Y, i.e., $Y^{X}$. Each pure strategy of the defender corresponds to a *deterministic* channel. Similarly, a *behavioural* strategy of the defender corresponds to a probabilistic channel, p(Y|X), whose space is ${\left(\Delta Y\right)}^{X}$. The adversary, after observing *y*, makes a guess *a* from the countable (but potentially infinitely-sized) set A. Hence, the space of the adversary’s pure strategies (deterministic plans of action) is $A^{Y}$. A behavioural strategy of the adversary, designated by q(A|Y), assigns a potentially probabilistic guess to each output. Hence, the space of adversary’s behavioural strategies is ${\left(\Delta A\right)}^{Y}$. A pure and behavioural *strategy profile* of the game are respectively the pairs $\left(d,a\right)\in \left(Y^{X}\times A^{Y}\right)$ and $\left(p\left(Y|X\right),q\left(A|Y\right)\right)\in \left({\left(\Delta Y\right)}^{X}\times {\left(\Delta A\right)}^{Y}\right)$.

The *payoff* of the game can in general be represented by the (bounded) function v:X×Y×A→R. That is, the outcome of each instance of the game is that the adversary wins, and the defender loses, v(x,y,a) units; if the (realisations) of the channel input, the channel output and the adversary’s guess have been *x*, *y*, and *a*, respectively. Let *V* represent the expected payoff of the game. The expectation is taken with respect to the random realisation of the input according to the prior *p* as well as any randomisation present in the strategies of the two players. Specifically,

(9) $$\begin{array}{c} V=\sum_{x\in X}\sum_{y\in Y}\sum_{a\in A}p\left(x\right)p\left(y|x\right)q\left(a|y\right)v\left(x,y,a\right). \end{array}$$

The defender wants to minimise *V* while the adversary wants to maximise it. Unlike the defender, the adversary does not observe the realisation of *X*; for this reason, this is a game of asymmetric information.

### 5.1. Nash Equilibria and Saddle-Point Strategies

A Nash equilibrium (NE) is a standard solution concept in game theory, which states that each strategy should be the best response assuming the strategy of the other player(s) is fixed. For two-player zero-sum games (2PZSGs), the set of NEs has a stronger interpretation—that of a *saddle point*. We first briefly describe this solution concept.

The defender may adopt the following worst-case scenario argument: assuming that any strategy that is adopted by the defender is going to be revealed to the adversary to best respond to it, the “robust” optimisation of the defender (the minimiser) becomes the following:

$$\begin{array}{c} \bar{V}≜\underset{p(Y|X)\in \Gamma }{\inf}\;\underset{q(A|Y)}{\sup}V\left(p(Y|X),q(A|Y)\right). \end{array}$$

We denote the value of the above optimisation with $\bar{V}$ to indicate that this is the highest expected payoff to the adversary. On the other hand, the best-case scenario of the defender is derived from the following argument: suppose the strategy of the adversary is given and the defender can design their strategy accordingly. Then this optimistic scenario for the defender (which is the worst-case for the adversary) leads to the following problem:

$$\begin{array}{c} \underline{V}≜\underset{q(A|Y)}{\sup}\;\underset{p(Y|X)\in \Gamma }{\inf}\;V\left(p(Y|X),q(A|Y)\right). \end{array}$$

Clearly, we have $\underline{V}\leq \bar{V}$. If we have $\underline{V}=\bar{V}=V^{*}$, we say the game has a *value* $V^{*}$. Further, a saddle-point strategy pair $\left(p^{*}\left(Y|X\right),q^{*}\left(X|Y\right)\right)$ is a strategy pair that satisfies the following:

$$\begin{array}{c} \forall p\left(X|Y\right)\in \Gamma ,\;V\left(p\left(Y|X\right),q^{*}\left(A|Y\right)\right)\leq V\left(p^{*}\left(Y|X\right),q^{*}\left(A|Y\right)\right)\leq V\left(p^{*}\left(Y|X\right),q^{*}\left(A|Y\right)\right),\;\forall q\left(A|Y\right)\in {\left(\Delta A\right)}^{Y}. \end{array}$$

That is, a saddle-point strategy attains the value of the game: $V^{*}=V\left(p^{*}\left(Y|X\right),q^{*}\left(A|Y\right)\right)$. Then the argument for the saddle-point strategies as the solution concept of the 2PZSG is strong: the saddle-point strategy gives each player a guarantee of the utility no-matter what the other player’s strategy is. In what follows, we derive the condition for the saddle-point strategy of the defender and adversary, respectively.

For the defender, a saddle-point strategy solves $\inf_{p(Y|X)\in \Gamma }\;\sup_{q(A|Y)}V\left(p(Y|X),q(A|Y)\right)$. As before, let $Y^{+}$ be the set of outputs with a strictly positive probability of realisation. Since only these “on-path” outputs contribute to the expected payoff, we can rewrite Equation (9) as

$$\begin{array}{c} V=\sum_{x\in X}\sum_{y\in Y^{+}}\sum_{a\in A}p\left(x\right)p\left(y|x\right)q\left(a|y\right)v\left(x,y,a\right)=\sum_{y\in Y^{+}}p\left(y\right)\sum_{a\in A}q\left(a|y\right)\sum_{x\in X}v\left(x,y,a\right)\frac{p(x)p(y|x)}{p(y)}\;\;\\ =\sum_{y\in Y^{+}}p\left(y\right)\sum_{a\in A}q\left(a|y\right)\sum_{x\in X}v\left(x,y,a\right)p\left(x|y\right). \end{array}$$

Hence,

$$\begin{array}{c} \underset{q(A|Y)}{\sup}V\left(p(Y|X),q(A|Y)\right)=\sum_{y\in Y^{+}}p\left(y\right)\underset{a\in A}{\sup}\sum_{x\in X}v\left(x,y,a\right)p\left(x|y\right). \end{array}$$

In particular, for each *y*, the adversary can put all the probability weight on an action that maximises the expected value of v(X,y,a) with $X∼p_{X|y}$, where $p_{X|y}$ follows Bayes’ rule. Note that, although we started from an agnostic stance, Bayes’ rule turns out to be indeed the optimal belief update of the adversary. The saddle-point strategy of the defender hence solves the following optimisation:

(10) $$\begin{array}{c} \underset{p(Y|X)\in \Gamma }{\inf}\sum_{y\in Y^{+}}p\left(y\right)\underset{a\in A}{\sup}\sum_{x\in X}v\left(x,y,a\right)p\left(x|y\right). \end{array}$$

For the saddle-point of the adversary, we can rewrite Equation (9) as

$$\begin{array}{c} V=\sum_{x\in X}p\left(x\right)\sum_{y\in Y^{+}}p\left(y|x\right)\sum_{a\in A}q\left(a|y\right)v\left(x,y,a\right). \end{array}$$

Therefore, the best strategy of the defender for a given *x* is to put all the probability weight of p(y|x) of the *y* that achieves the smallest $\sum_{a\in A}q\left(a|y\right)v\left(x,y,a\right)$ across all feasible *y*’s for that *x*, i.e.,

$$\begin{array}{c} \underset{p(Y|X)}{\inf}V\left(p(Y|X),q(A|Y)\right)=\sum_{x\in X}p\left(x\right)\underset{y\in Y:(x,y)\in \Omega }{\inf}\sum_{a\in A}v\left(x,y,a\right)q\left(a|y\right). \end{array}$$

Hence, the saddle-point strategy of the adversary comes from solving the following optimisation:

(11) $$\begin{array}{c} \underset{q\left(A|Y\right)\in {\left(\Delta A\right)}^{Y}}{\sup}\sum_{x\in X}p\left(x\right)\underset{y\in Y,(x,y)\in \Omega }{\inf}\sum_{a\in A}v\left(x,y,a\right)q\left(a|y\right). \end{array}$$

We will consider the following payoff function:

$$\begin{array}{c} v(x,y,a)=g(a,x)-\lambda u(x,y) \end{array}$$

where $\lambda \in R^{+}$ and g,u are real valued functions. This payoff function can be understood as a weighted difference between the gain of the attacker in guessing the secret and the utility of the channel.

We will refer to such zero-sum game between a defender and an adversary as G (also *G*-game), which is specified by 〈p,Ω,g(x,a),λ,u(x,y)〉. For such a game, the optimisation problem for saddle-point strategy of the defender in Equation (10) becomes

(12) $$\begin{array}{c} \underset{p(Y|X)\in \Gamma }{\inf}\left\{\sum_{y\in Y^{+}}p\left(y\right)\underset{a\in A}{\sup}\left(\sum_{x\in X}g\left(x,a\right)p\left(x|y\right)-\lambda \sum_{x\in X}p\left(x|y\right)u\left(x,y\right)\right)\right\}\\ =\underset{p(Y|X)\in \Gamma }{\inf}\left\{\sum_{y\in Y^{+}}p\left(y\right)\underset{a\in A}{\sup}\left(\sum_{x\in X}g\left(x,a\right)p\left(x|y\right)\right)-\lambda \sum_{x,y}p\left(x\right)p\left(y|x\right)u\left(x,y\right)\right\}. \end{array}$$

**Theorem** **1.** *For any optimal channel design problem in Equation *(7)*, there is an induced game G, where the optimal channel is the saddle-point strategy of the defender. Conversely, for any game G, the saddle-point strategy of the defender is a solution to an induced optimal channel design problem*.

**Proof.**  We showed in Proposition 3 that the optimal channel design problem for any core-concave *H* is a convex optimisation. Since η is an increasing function, it can be removed from the optimisation without any effect. Now, from convex optimisation theory, we know that there exists a Lagrange multiplier λ≥0 such that the solutions of the original optimisation matches those of the following Lagrange relaxation problem:

$$\begin{array}{c} \underset{p_{Y|X}\in \Gamma }{\sup}\left\{\sum_{y\in Y^{+}}p\left(y\right)F\left(p_{X|y}\right)+\lambda \left(\sum_{x,y}p\left(x\right)p\left(y|x\right)u\left(x,y\right)-u_{\min}\right)\right\}. \end{array}$$

Or equivalently,

$$\begin{array}{c} -\underset{p_{Y|X}\in \Gamma }{\inf}\left\{\sum_{y\in Y^{+}}p\left(y\right)\left(-F\left(p_{X|y}\right)\right)-\lambda \sum_{x,y}p\left(x\right)p\left(y|x\right)u\left(x,y\right)\right\}+\lambda u_{\min}. \end{array}$$

Now, since −F(p) is a convex function of p∈ΔX, there is a countable set A and a function $g_{F}:A\times X\to R$ such that

$$\begin{array}{c} \forall p\in \Delta X,\;-F\left(p\right)=\underset{a\in A}{\sup}\sum_{x\in X}p\left(x\right)g_{F}\left(a,x\right). \end{array}$$

In particular, $g_{F}\left(a,x\right)$ can be constructed as follows: This follows from application of the supporting hyperplanes and a limit argument, as presented, e.g., in [19] (Theorem 5). Therefore, the optimisation can be written as

$$\begin{array}{c} -\underset{p(Y|X)\in \Gamma }{\inf}\left\{\sum_{y\in Y^{+}}p\left(y\right)\left(\underset{a\in A}{\sup}\sum_{x\in X}p\left(x|y\right)g_{F}\left(a,x\right)\right)-\lambda \sum_{x,y}p\left(x\right)p\left(y|x\right)u\left(x,y\right)\right\}+\lambda u_{\min}. \end{array}$$

Now, note that the minimisation is defining exactly the saddle-point strategy of the defender in a game $G=\langle p,\Omega ,g_{F}\left(x,a\right),\lambda ,u\left(x,y\right)\rangle$ as given in Equation (12).

Now, for the reverse direction, consider the game G=〈p,Ω,g(x,a),λ,u(x,y)〉. The saddle-point strategy of the defender is a solution of the optimisation in Equation (12). Note that the $\sup_{a\in A}\sum_{x\in X}p\left(x|y\right)g\left(a,x\right)$ characterises a convex function, or a negative of a concave function, which we call $F_{g}$, i.e., let

$$\begin{array}{c} -F_{g}\left(p_{X|y}\right)≜\underset{a\in A}{\sup}\sum_{x\in X}p\left(x|y\right)g\left(a,x\right). \end{array}$$

With this notation, the saddle-point strategy of the defender solves

(13) $$\begin{array}{c} \underset{p(Y|X)\in \Gamma }{\inf}-\left\{\sum_{y\in Y^{+}}p\left(y\right)F_{g}\left(p_{X|y}\right)-\lambda \sum_{x,y}p\left(x\right)p\left(y|x\right)u\left(x,y\right)\right\}. \end{array}$$

Let a saddle-point strategy of the defender be denoted by $p^{*}\left(Y|X\right)$. Now consider the the following convex optimisation:

(14) $$\begin{array}{c} -\underset{p(Y|X)\in \Gamma }{\inf}\sum_{y\in Y^{+}}p\left(y\right)F_{g}\left(p_{X|y}\right)\;s.t.\;\sum_{x,y}p\left(x\right)p\left(y|x\right)u\left(x,y\right)\geq u_{\min} \end{array}$$

where $u_{\min}=\sum_{x,y}p\left(x\right)p^{*}\left(y|x\right)u\left(x,y\right)$. We claim that these two convex optimisations are equivalent. To see this, note that the KKT conditions are necessary and sufficient for optimality in both optimisations. Moreover, if we take the λ in Equation (13) to be the Lagrange multiplier of the minimum utility constraint in Equation (14), these KKT conditions are exactly identical, except that Equation (14) has an additional complementary slackness condition: $\lambda (\sum_{x,y}p\left(x\right)p\left(y|x\right)u\left(x,y\right)-u_{\min})=0$. Since λ>0, we should have, for an optimum of Equation (14), $\sum_{x,y}p\left(x\right)p\left(y|x\right)u\left(x,y\right)-u_{\min}=0$, which holds for the saddle-point strategy by our specific choice of $u_{\min}=\sum_{x,y}p\left(x\right)p^{*}\left(y|x\right)u\left(x,y\right)$. ☐

When the action-space of the adversary is finite, the saddle point strategies can be computed using linear programming. Specifically:

**Proposition** **5.** *If the game has a finite number of pure strategies, then the saddle point strategies expressed by Equations *(10)* and *(11)* can be computed as the solution to the following linear program (LP) and its dual:*

$$\begin{array}{c} {\bar{V}}^{*}=\min_{p,v}\sum_{y\in Y}v_{y}-\lambda \sum_{x,y}p\left(x\right)p\left(y|x\right)u\left(x,y\right)\\ s.t.:\;v_{y}\geq \sum_{x\in X}g\left(a,x\right)p\left(x\right)p\left(y|x\right),\;\forall a\in A,\;\forall y\in Y\\ p(y|x)\geq 0,\forall y\in Y,\forall x\in X,\;\sum_{y\in Y}p\left(y|x\right)=1,\;\forall x\in X,\;p\left(y|x\right)=0,\forall \left(x,y\right)\notin \Omega . \end{array}$$

*Introducing variables $u=(u_{x})$ for x∈X, the dual of the above LP is*

$$\begin{array}{c} {\underline{V}}^{*}=\max_{\alpha ,u}\sum_{x\in X}p\left(x\right)u_{x}\\ s.t.:\;u_{x}\leq \sum_{a\in A}\left(g(a,x)-\lambda u(x,y)\right)q\left(a|y\right),\;\forall (x,y)\in \Omega \\ q(a|y)\geq 0,\;\forall a\in A,\forall y\in Y,\;\sum_{a\in A}q\left(a|y\right)=1,\;\forall y\in Y. \end{array}$$

**Proof.**  In the first LP, the constraints $v_{y}\geq \sum_{x\in X}g\left(a,x\right)p\left(x\right)p\left(y|x\right),\;\forall a\in A,$ and ∀y∈Y guarantee that, for each *y*, the optimisation chooses $v_{y}=\max_{a\in A}\sum_{x\in X}g\left(a,x\right)p\left(x\right)p\left(y|x\right)$; hence, the objective function becomes exactly as in Equation (10).

Similarly, for the second LP, the constraints $u_{x}\leq \sum_{a\in A}\left(g(a,x)-\lambda u(x,y)\right)q\left(a|y\right),$ and ∀(x,y)∈Ω guarantee that the optimisation chooses $u_{x}=\min_{y\in Y,(x,y)\in \Omega }\sum_{a\in A}\left(g(a,x)-\lambda u(x,y)\right)q\left(a|y\right)$, which is exactly the optimisation problem of the adversary as in Equation (11). ☐

### 5.2. The Adversary’s Problem: Robust Inference

One important advantage of the game-theoretical analysis is that it connects the problem of the defender and attacker. Here, we provide a practical interpretation of the adversary’s problem: Suppose we would like to extract information (i.e., infer) about *X* by observing *Y*. We know the prior over *X*, but we do not know $p_{Y|X}$, i.e., the channel. All we know is that the channel has to respect some hard and/or soft operational constraints. What is the best inference about *X* in the absence of the channel? One approach is to consider the worst case among all possible channels that satisfy the constraints. The resulting “robust” strategy will have the minimum inference guarantee for any feasible realisation of the channel. The game-theoretical analysis reveals that the optimal channel design problem and the robust inference problem are equivalent; i.e., they are duals of each other.

### 5.3. Measure-Invariant Optimality

Notice that in all cases seen so far the optimal solution depends on the choice of entropy. There is, however, a particular case studied in Reference [1] where the optimiser is universal, i.e., is the same for all entropies:

**Proposition** **6.** *(Theorem 1 in Reference [1]) When there are no soft constraints and the hard constraints are equivalent to just a size-cap of k on the pre-images of the outputs, there is a closed form solution for the Nash equilibrium. Moreover, this solution is universally optimal, i.e., it is optimal for any choice of entropy*.

## 6. Uncertainty about the Prior

We have assumed that the input is realised according to a single distribution *p* that is known to the adversary. We now analyse the setting where the prior distribution of the input can be one of a number of possibilities, each happening with a known probability (a distribution over distributions. That is, the distribution of the input itself depends on a hidden random variable, which we refer to as the *context*. The adversary knows the joint statistics of the hidden context and the input, but does not get to observe the realisation of the context.

At a high level, the main result of this section is the following: the best strategy for the defender is *not* to “customise” its strategy with respect to the context depending on the particular prior given each context, but rather to build an “averaged prior”, and design the best strategy over this averaged prior and play it irrespective of the contexts. This result implies that the context-dependent optimal channel design problem reduces to an equivalent context-independent channel design problem over the mixed prior.

This result may not be immediately intuitive, as there can be a counterargument as follows: Among the available priors, there are some particularly “good” ones, in the sense that they are very conducive to hide the secret (e.g., they are very close to uniform in a symmetric constraint setting). Then shouldn’t the defender adopt the optimal channel for such priors in those contexts—especially if they have a high probability weight of occurrence? Our result refutes this intuitive argument.

To formalise the setting, let the space of the discrete random variable of the context be $C=\{c_{1},\ldots ,c_{\left|C\right|}\}$. Without loss of generality, we assume that the context has full support, i.e., $p_{C}\left(c\right)>0,\;\forall c\in C$. The channel designer (the defender) knows the true distribution of the secret. Technically speaking, the defender “observes” the realisation of the context. The adversary, on the other hand, does not directly observe the context, but knows the probability of the realisation of each context, $p_{C}$, as well as the (conditional) probability distribution of the secret given each context, $p_{X|C}$. Note that knowledge of $p_{C}$ and $p_{X|C}$ is equivalent to the knowledge of the “joint” probability distribution of the context and the secret $p_{X,C}$.

The adversary only sees the output *Y* and wants to “infer” about the input *X*. In the worst case, one can assume that the adversary knows $p_{Y|X,C}$ and hence, using his knowledge of $p_{X,C}$, can use Bayes’ rule to update his best belief about the secret after observing *Y*, i.e., constructing his *posterior*:

$$\begin{array}{c} p\left(x|y\right)=\frac{p(x,y)}{p(y)}=\frac{\sum_{c\in C}p\left(c\right)p\left(x|c\right)p\left(y|x,c\right)}{\sum_{x^{\prime }\in X}\sum_{c\in C}p\left(c\right)p\left(x^{\prime }|c\right)p\left(y|x^{\prime },c\right)}. \end{array}$$

Note that the defender is not directly interested in not leaking information about the context and only cares about *X*, but should be wary of how the adversary can use their information about the joint distribution of the context and input to intuit the input based on the observation. In addition, for clarity, we repeat that the adversary does not “observe” the context nor the secret. (For the scenario where the adversary can directly observe the context, the problem will reduce to designing |C| optimal channels according to optimisations as in Equation (7) with priors $p_{X|c}$ for each c∈C.)

The defender decides what observable to produce per each secret in each context, potentially using randomisation and benefit from the ambiguity that it can inject. As before, the strategy has to satisfy some operational constraints. We may have hard constraints prescribing which secrets can produce which observables, which in part determine which subsets of secrets can be conflated with each other. In the previous sections, we expressed these “hard” operational constraints through Ω⊆X×Y, representing the set of permissible secret-observable pairs. In the presence of contexts, in the most general form, the permissible observables for a secret may depend on the context as well; thus, Ω should be now a subset of X×C×Y. However, for the result of this section, we assume that these constraints are context-independent, i.e., the same subset of observables is permissible for a secret irrespective of the context, so we keep Ω to be a subset of X×Y.

Likewise, there can be soft operational constraints in the form of satisfying a minimum expected utility. The expectation is now taken with respect to the context as well, that is, we must have expectation of the payoff with respect to X,C,Y to be no less than $u_{\min}$. However, for the result of this section, we assume that the payoff function *u*, i.e., the measure of “goodness” of each observable for each secret, does not depend on the context. Hence,

$$\begin{array}{c} \sum_{x,c,y}p\left(x,c\right)p\left(y|x,c\right)u\left(x,y\right)\geq u_{\min}. \end{array}$$

As before, without loss of generality, assume that we are dealing with core-concave functions, i.e., *F* is concave and η is increasing. Moreover, note that, again, the choice of the strategy cannot affect the prior entropy of the secret. Hence, the problem of designing for minimum leakage is again equivalent to maximising the posterior entropy. Ignoring η, since it is just an increasing scalar function, the posterior entropy (as the objective of the maximisation) can hence be written as

(15) $$\begin{array}{c} \max_{p_{Y|X,C}}\sum_{y\in Y^{+}}p\left(y\right)F\left(p_{X|y}\right),\;\mathrm{where}\;p\left(y\right)=\sum_{x,c}p\left(x,c\right)p\left(y|x,c\right),\;\mathrm{and}\;p\left(x|y\right)=\frac{\sum_{c}p\left(x,c\right)p\left(y|x,c\right)}{p(y)}. \end{array}$$

The constraints of the optimisation are:

(16a) $$\begin{array}{c} p(y|x,c)\geq 0\;\;\;\;\;\;\forall y\in Y,\;(x,c)\in X\times C \end{array}$$

(16b) $$\begin{array}{c} \sum_{y\in Y}p\left(y|x,c\right)=1\;\;\;\;\;\;\;\forall (x,c)\in X\times C \end{array}$$

(16c) $$\begin{array}{c} p(y|x,c)=0\;\;\;\;\;\;\;\;\;\;\;\;(x,y)\notin \Omega \end{array}$$

(16d) $$\begin{array}{c} \sum_{x,c,y}p\left(x,c\right)p\left(y|x,c\right)u\left(x,y\right)\geq u_{\min}. \end{array}$$

Given any “context-dependent” strategy *p*, we define a corresponding “context-independent” strategy $\bar{p}$ as follows:

(17) $$\begin{array}{c} \bar{p}\left(y|x\right)=\sum_{c}p\left(c|x\right)p\left(y|x,c\right). \end{array}$$

To be precise, the strategy is $\tilde{p}$ such that for any $c^{\prime }\in C$, $\tilde{p}\left(y|x,c^{\prime }\right)=\bar{p}\left(y|x\right)$, i.e., $\tilde{p}$ represents playing the same randomised strategy of $\bar{p}$ irrespective of the context. This context-free strategy is a mixing of the context-dependent strategies with weights equal to conditional probability of the context given the secret. In other words, $\tilde{p}$ “marginalises away” the dependence of *p* on the context. Note however, that we cannot marginalise away the dependence on *X*, because of the input-dependent constraints: these input-dependent constraints are exactly why the trivial solutions like p(y|x,c)=1/∥Y∥ are not acceptable.

First, we show that $\tilde{p}$ is itself a legitimate strategy:

1. $\tilde{p}\left(y|x,c\right)\geq 0$: trivially (product of non negative terms).
2. $\forall \left(x,c\right)\in X\times C:\;\sum_{y\in Y}\tilde{p}\left(y|x,c\right)=1$; this is because
  $$\begin{array}{c} \sum_{y}\tilde{p}\left(y|x,c\right)=\sum_{y}\sum_{c^{\prime }}p\left(c^{\prime }|x\right)p\left(y|x,c^{\prime }\right)=\sum_{c^{\prime }}p\left(c^{\prime }|x\right)\sum_{y}p\left(y|x,c^{\prime }\right)=\sum_{c^{\prime }}p\left(c^{\prime }|x\right)=1 \end{array}$$
  where we first exchanged the order of the summations, and then respectively used the facts that $p(y|x,c^{\prime })$ and $p(c^{\prime }|x)$ are conditional distributions.
3. We show that the expected payoff under strategy $p_{Y|X,C}$ is the same as the expected payoff under strategy ${\tilde{p}}_{Y|X,C}$. Therefore, if $p_{Y|X,C}$ satisfies the minimum expected payoff constraint, so does ${\tilde{p}}_{Y|X,C}$. For this purpose, we establish the following lemma, which we will user later:
  **Lemma** **1.** *Let $p_{X,Y}$ and ${\tilde{p}}_{X,Y}$ denote the induced (joint) distribution on X×Y where, respectively, strategies $p_{Y|X,C}$ and ${\tilde{p}}_{Y|X,C}$ are employed. Then we have $p\left(x,y\right)=\tilde{p}\left(x,y\right)$∀x,y∈X×Y*.
  **Proof.**  $p\left(x,y\right)=\sum_{c}p\left(c|x\right)p\left(y|x,c\right)=\bar{p}\left(y|x\right)=\bar{p}\left(y|x\right)\sum_{c}p\left(c|x\right)=\sum_{c}p\left(c|x\right)\tilde{p}\left(y|x,c\right)=\tilde{p}\left(x,y\right)$. ☐
  Now, the equality of the expected payoff under these two strategies follows as a simple corollary:
  $$\begin{array}{c} \sum_{x,c,y}p\left(x,c\right)p\left(y|x,c\right)u\left(x,y\right)=\sum_{x,c,y}p\left(x,y,c\right)u\left(x,y\right)=\sum_{x,y}p\left(x,y\right)u\left(x,y\right)=\sum_{x,y}\tilde{p}\left(x,y\right)u\left(x,y\right). \end{array}$$
  The second equality holds because u(x,y) does not depend on *c*, and $\sum_{c}p\left(x,y,c\right)=p\left(x,y\right)$. The third equality is due to Lemma 1.
4. $\tilde{p}\left(y|x,c\right)=0$$\forall \left(y,x\right)\notin \Omega_{x}$, trivially. Note that we made the assumption that the hard constraints do not depend on the context, and only on the input.

Next, we show that replacing any context-dependent strategy with its context-independent counterpart would lead to same leakage (irrespective of the choice of the entropy).

**Lemma** **2.** *Let H(X|Y) and $\tilde{H}\left(X|Y\right)$ denote the posterior entropies where strategies $p_{Y|X,C}$ and its corresponding ${\tilde{p}}_{Y|X,C}$ are used. Then we have $H\left(X|Y\right)=\tilde{H}\left(X|Y\right)$*.

**Proof.**  This is also a direct consequence of Lemma 1, once we notice that H(X|Y) is completely determined by $p_{X,Y}$. ☐

This in turn implies that the search for optimal channels can be restricted to the context-independent ones. We are now ready for the main result of this section: that the (informally) optimal channel problem with uncertainty can be reduced to the classical case of Equation (7):

**Theorem** **2.** *The optimisation in Equation *(15)* subject to Equation *(16)* can be simplified to an instance of Equation *(7)* where the prior distribution is the context-average prior, i.e., $\sum_{c\in C}p\left(c\right)p_{X|c}$. In particular, if $p_{Y|X}^{*}$ is an optimal solution of Equation *(7)* with the average prior, then an optimal solution of Equation *(15)* subject to Equation *(16)* is to play $p_{Y|X}^{*}$ for all c∈C*.

**Proof.**  This proof follows a similar argument as above. In particular, if we let $\tilde{p}\left(y|x,c\right)=p^{*}\left(y|x\right)$ for all c∈C, the constraints of Equations (16a)–(16c) follow directly from feasibility of $p_{Y|X}^{*}$ for Equation (7). Now, let the joint probability on X×Y induced by ${\tilde{p}}_{Y|X,C}$ and $p_{Y|X}^{*}$ be respectively denoted by ${\tilde{p}}_{X,Y}$ and $p_{X,Y}^{*}$. Then $\tilde{p}\left(x,y\right)=\sum_{c}p\left(c\right)p\left(x|c\right)\tilde{p}\left(y|x,c\right)=\sum_{c}p\left(c\right)p\left(x|c\right)p^{*}\left(y|x\right)$. On the other hand, $p^{*}\left(x,y\right)=p\left(x\right)p^{*}\left(y|x\right)$, where p(x) is the prior used in Equation (7). Hence, by taking this prior to be $\sum_{c}p\left(c\right)p\left(x|c\right)$, we ensure that ${\tilde{p}}_{X,Y}=p_{X,Y}^{*}$. This in turn implies that ${\tilde{p}}_{Y|X,C}$ satisfies Equation (16d) and, further, has the same H(X|Y) as of $p^{*}\left(y|x\right)$, which by construction has the highest value. Finally, from Lemma (2), H(X|Y) is also the highest value across all (potentially context-dependent) channels. ☐

### 6.1. Game-Theoretical Interpretation

Let us consider now the implications of Theorem 2 with respect to our game-theoretical interpretation: Notice first we can cast the uncertainty on the prior in terms of a Bayesian game over G-games as defined in Section 5: Nature chooses one of the possible priors, and the players then play in the G-game corresponding to that prior. Theorem 2 says that the defender optimal strategy in this Bayesian game is to play the Nash equilibrium strategy from the G-game corresponding to the average prior.

The adversary has to best respond to the defender move (as the defender plays first), and, as the attacker does not know the subgame chosen by Nature but only sees the move played by the defender (all sub-games in the Bayesian game have the same set of moves), he can only best respond over the average prior.

Hence, the Nash equilibrium in the Bayesian game over a set of priors is given by the Nash equilibrium over the G-game over the average prior specified in Theorem 2.

### 6.2. Discussion

**Figure 3:**
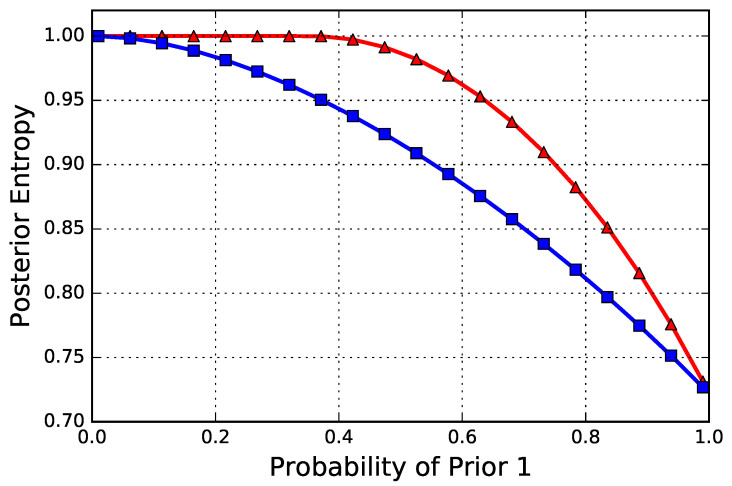
Shannon’s posterior entropy between the optimal design as per Proposition 2 and the heuristic best alternative, where the best channel for each prior is designed and played according to the context. The priors are as follows: $P_{1}=\left(1/3,1/3,1/3\right)$ (the “good” prior) and $P_{2}=\left(0.8,0.15,0.05\right)$ (the “bad” prior). The *x*-axis is the probability (weight) of P1. As we can see, except trivially for the two end-points, the optimal strictly outperforms this “best” heuristic.

As mentioned in the beginning of this section, an alternative heuristic is to play the best channel per each context. One can argue that, if the “good” priors that lead to a particularly strong channel have a high probability, it may be better to play this heuristic. However, as we established in Theorem 2, this heuristic is wrong. For a numerical depiction, in Figure 3, we have plotted the posterior entropy that is achieved by the optimal strategy $\bar{p}$ per Theorem 2 against this heuristic strategy of playing the best channel for each prior. As we can see, for any weight of the two priors (except trivially when the weight is either 0 or 1, where the two strategies become the same), the $\bar{p}$ strictly outperforms the heuristic strategy.

## 7. Conclusions and Future Work

We investigated the problem of designing constrained channels that leak minimally about their input in a general information-theoretical setting. We generalised the notion of information leakage that encompassed a broad range of existing entropy-based measures and established that with respect to all of such measures, the problem of designing optimal channels is a convex optimisation with zero duality gap, where KKT conditions provide both necessary and sufficient conditions of optimality.

We then introduced a game-theoretical framework in which the channel designer is a defender against an information extracting adversary, and showed that the Bayes–Nash equilibrium strategies of the defender correspond to the optimal channels. The game-theoretical framework reveals a dual connection between our optimal channel design and a robust inference problem. In particular, the equilibrium strategies of the adversary solve the following interesting problem: Suppose we know the prior distribution of a random variable and the operational specification of a channel in terms of soft and hard constraints but the exact realisation of the channel is not known, and we would like to make the best inference about the input by observing the output of an instance of such channels. In particular, the equilibrium strategies of the adversary are robust, in the sense that they guarantee a minimal level of inference for any realisation of the channel within the family of the given constraints.

While in this work we emphasised the viewpoint of the defender, future work can investigate the adversary’s problem of robust inference further. Moreover, as suggested by one reviewer, the implication of our results to a general system design and analysis, for instance in the sense of Žampa’s systems theory [27], will be an interesting trajectory. This is inspired by the observation that our notion of a “channel” can be seen as an example of a “stochastic (abstract) system”.

## Figures and Tables

**Table 1:** List of the main notations for the optimal channel design problem.

Parameter	Definition
X,Y	input and output random variables of the channel.
$p_{X}$	(given) prior distribution on the input of the channel.
Ω	(given) set of permissible input–output pairs (hard constraints).
u(x,y)	utility of the channel input–output pair is (x,y).
$u_{\min}$	minimum expected utility that the channel must satisfy (soft constraint).
$p_{Y|X}$	representation of the channel as the conditional distributions given each input.
$p_{X|y}$	(Bayesian) posterior distribution of the input if the observed output of the channel is *y*.
H(X)	(prior) entropy of the input, equal to $\eta (F\left(p_{X}\right))$ where η is increasing and *F* is concave.
H(X|Y)	posterior entropy of the input, equal to $\eta \left(\sum_{y}p\left(y\right)F\left(p_{X|y}\right)\right)$ for the same η and *F*.
Leakage	leakage of information about input by observing outputs, equal to H(X)−H(X|Y).
